# Author Correction: Spatially clustered loci with multiple enhancers are frequent targets of HIV-1 integration

**DOI:** 10.1038/s41467-021-26471-w

**Published:** 2021-10-28

**Authors:** Bojana Lucic, Heng-Chang Chen, Maja Kuzman, Eduard Zorita, Julia Wegner, Vera Minneker, Wei Wang, Raffaele Fronza, Stefanie Laufs, Manfred Schmidt, Ralph Stadhouders, Vassilis Roukos, Kristian Vlahovicek, Guillaume J. Filion, Marina Lusic

**Affiliations:** 1 Department of Infectious Diseases, Integrative Virology, Heidelberg University Hospital and German Center for Infection Research, Heidelberg, Germany; 2 Genome Architecture, Gene Regulation, Stem Cells and Cancer Programme, Center for Genomic Regulation (CRG), Barcelona Institute of Science and Technology, Barcelona, Spain; 3 University Pompeu Fabra, Barcelona, Spain; 4 Bioinformatics Group, Division of Molecular Biology, Department of Biology, Faculty of Science, University of Zagreb, Zagreb, Croatia; 5 Institute of Molecular Biology (IMB), Mainz, Germany; 6 German Cancer Research Center (DKFZ) and National Center for Tumor Diseases (NCT), Heidelberg, Germany; 7 Department of Pulmonary Medicine, Erasmus MC, Rotterdam, The Netherlands; 8 Department of Cell Biology, Erasmus MC, Rotterdam, The Netherlands; 9 Department of Biological Sciences, University of Toronto Scarborough, Toronto, ON Canada; 10 Present Address: Institute for Clinical Chemistry and Clinical Pharmacology, Universitätsklinikum Bonn, Bonn, Germany

**Keywords:** Computational biology and bioinformatics, Genetics, Retrovirus, Chromatin structure, Nuclear organization

Correction to: *Nature Communications* 10.1038/s41467-019-12046-3, published online 6 September 2019.

In this article the funding from the Deutsche Forschungsgemeinschaft (DFG, German Research Foundation) - Project number 240245660- SFB 1129, Project 20 was omitted. The original article has been corrected.

