# Supplementary material for: Spatially clustered loci with multiple enhancers are frequent targets of HIV-1 integration
Source: Nat Commun. 2019 Sep 6;10:4059. doi: 10.1038/s41467-019-12046-3 (PMC6731298; doi:10.1038/s41467-019-12046-3)
Supplement: Supplementary file 1 — Supplementary Information [file 41467_2019_12046_MOESM1_ESM.pdf]

NCOMMS-18-14402D
Supplementary information

Spatially clustered loci with multiple enhancers are frequent targets of HIV-1 integration

Lucic et al.

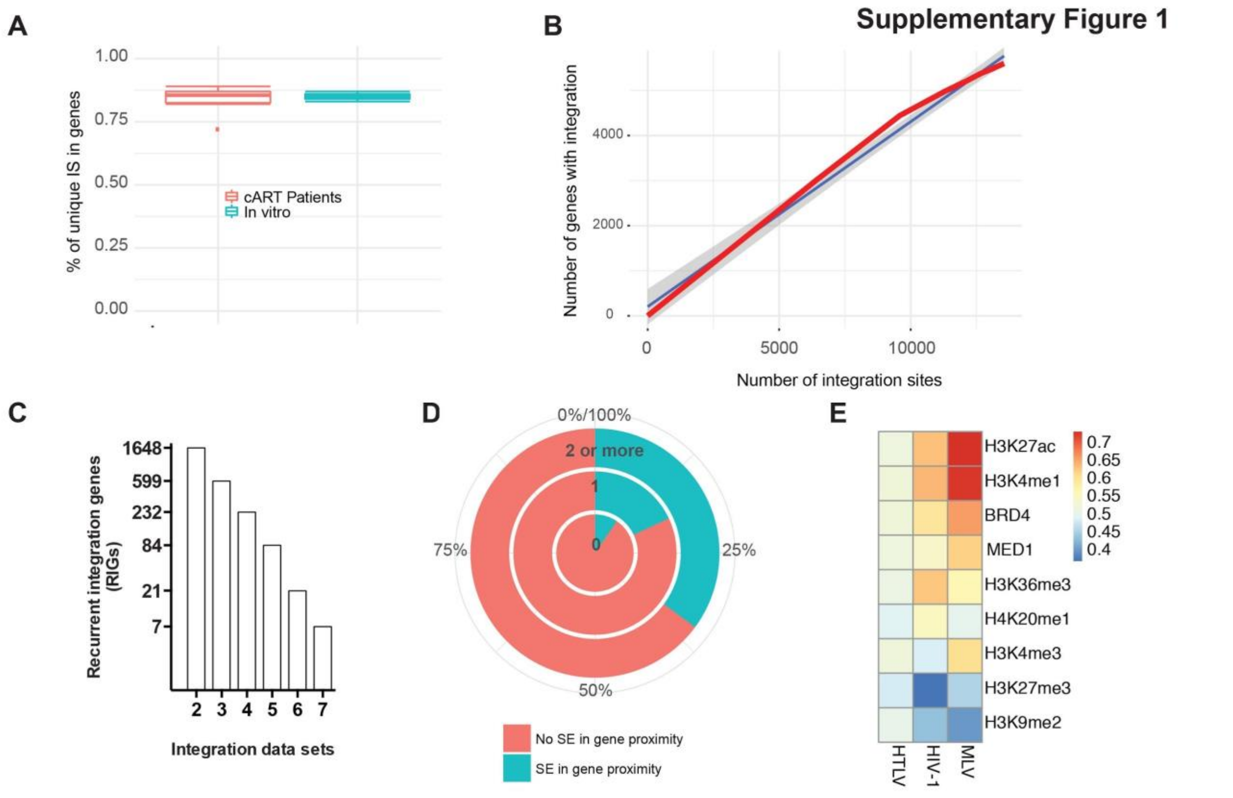

Supplementary Figure 1.

A) HIV-1 integration sites inside genes. The box plot represents the percentage of integration sites inside genes for cART-treated patients in rose (6 lists in total) and in vitro infections in blue (2 lists in total). The whiskers stretch from 5th to 95th percentile.

B) Analysis of the number of unique genes containing integration sites versus number of observed integrations. All integration data sets are sorted by decreasing size and the cumulative number of integration sites is plotted on the X-axis while the Y-axis shows the number of unique genes that have integrations. The number of unique genes found when analyzing different numbers of data sets linearly depends on the number of integrations in the observed data sets (adjusted R^2 = 0.9896, p = 2.075e^{-8}).

C) HIV-1 recurrently integrates into a subset of genes. The bar plot represents the number of genes (RIGs) shared among at least x different data sets. The number of RIGs shared among different data sets decreases exponentially as more data sets are taken into consideration.

D) Bar plot showing the percentage of genes that have a super-enhancer in proximity, in groups of genes on 0 list (without HIV-1 integrations), genes on 1 list and genes in 2 or more lists (RIGs).

E) ROC analysis represented as heatmap summarizing the co-occurrence density of integration sites and epigenetic modification obtained by ChIP-Seq for H3K27ac, H3K4me1, BRD4, MED1, H3K36me3, H4K20me1, H3K4me3, H3K27me3 and H3K9me2. HTLV**,** HIV-1 and MLV integration data sets are shown in the columns, and epigenetic modifications are shown in rows. Associations are quantified using the ROC area method; values of ROC areas are shown in the color key at the right.

Supplementary Figure 2

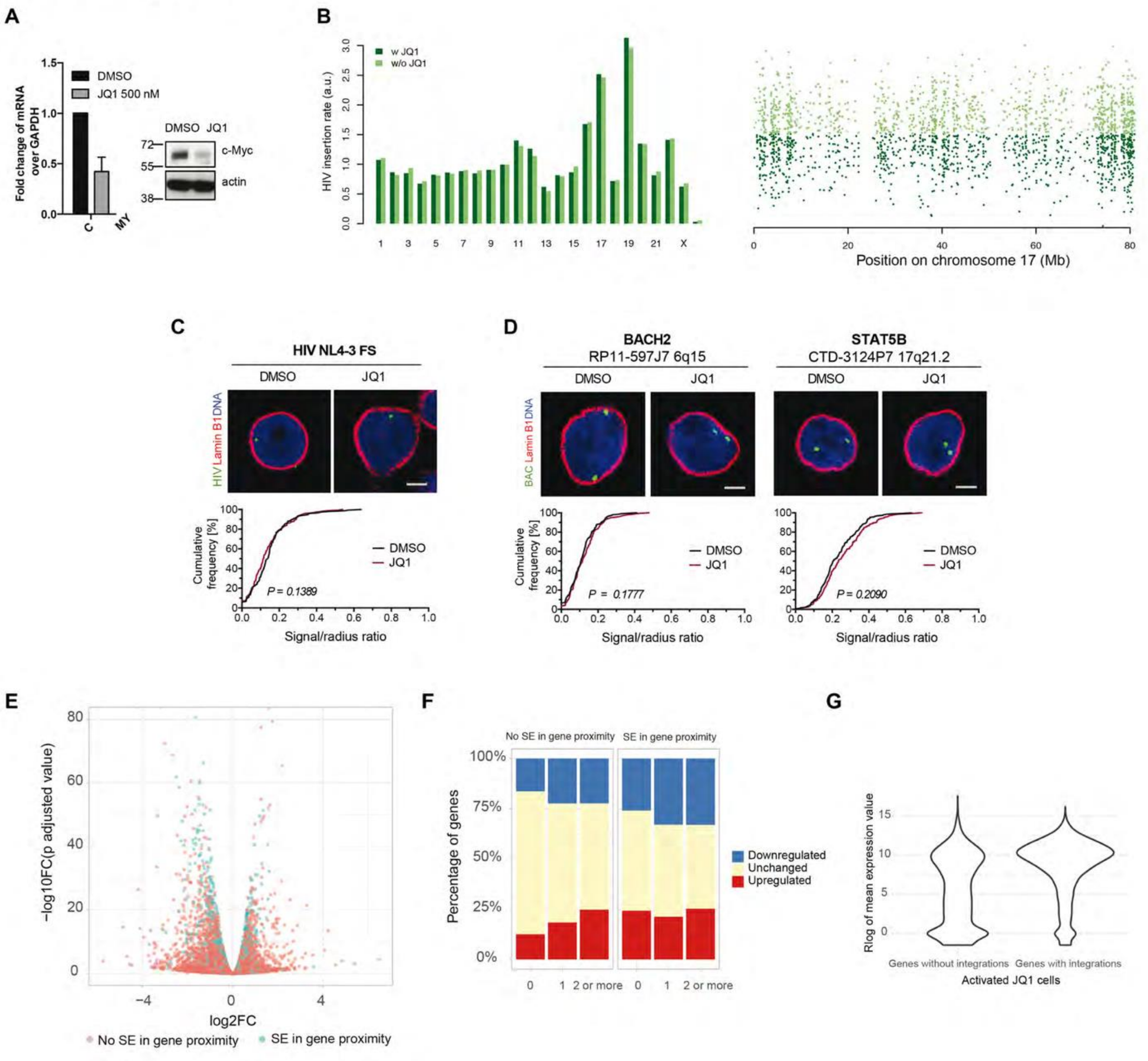

Supplementary Figure 2.

A) mRNA expression profiles and protein levels of MYC upon JQ1 treatment (500 nM JQ1 for 6 h). The mRNA levels are normalized over GAPDH, and mean and standard deviation are derived from three independent experiments. Representative protein levels of c-Myc and actin are shown on the western blot.

B) Bar plot of HIV-1 insertion rate per chromosome: control (w/o JQ1) or JQ1 treated (w JQ1) samples (left panel) and ‘insertion cloud’ representation on chromosome 17 with characteristic 3-fold enrichment (right panel). Each dot represents an HIV-1 insertion site. The x-coordinate indicates to the location of the insertion site on chromosome 17; the y-coordinate is random so that insertion hotspots appear as vertical lines. The insertion profile upon JQ1 treatment was flipped vertically

C) 3D immuno-DNA FISH images of HIV-1 in activated $CD4^+$ T cells pretreated with 500 nM JQ1 for 6 h and infected for 72 h (green: HIV-1 probe, red: lamin B1, blue: DNA staining with Hoechst 33342, scale bar represents 2 μm). Cumulative frequency plots show combined data

from both experiments (n = 100, black: DMSO, red: JQ1). The p-values of the Kolmogorov-Smirnov tests are indicated.

D) 3D immuno-DNA FISH images of *BACH2* and *STAT5B* upon 500 nM JQ1 treatment for 6 h in activated $CD4^+$ T cells (green: BAC/gene probe, red: lamin B1, blue: DNA staining with Hoechst 33342, scale bar represents 2 μm).

Regularized log transformed read counts on protein coding genes averaged over three replicates in activated JQ1 treated cells shown as violin plot for genes grouped by presence of HIV-1 integration in activated JQ1 treated cells.

E) Volcano plot showing the changes in mRNA levels of protein coding genes upon JQ1 treatment with respect to the vicinity to super-enhancers.

F) Bar plot showing the percentage of protein coding genes that are downregulated, unchanged, and upregulated upon JQ1 treatment. Genes are grouped by number of lists they occur in and by the presence or absence of super-enhancer in either gene body or 5 kb upstream.

G) Regularized log transformed read counts on protein coding genes averaged over three replicates in activated JQ1 treated cells shown as violin plot for genes grouped by presence of HIV-1 integration in activated JQ1 treated cells.

Supplementary Figure 3

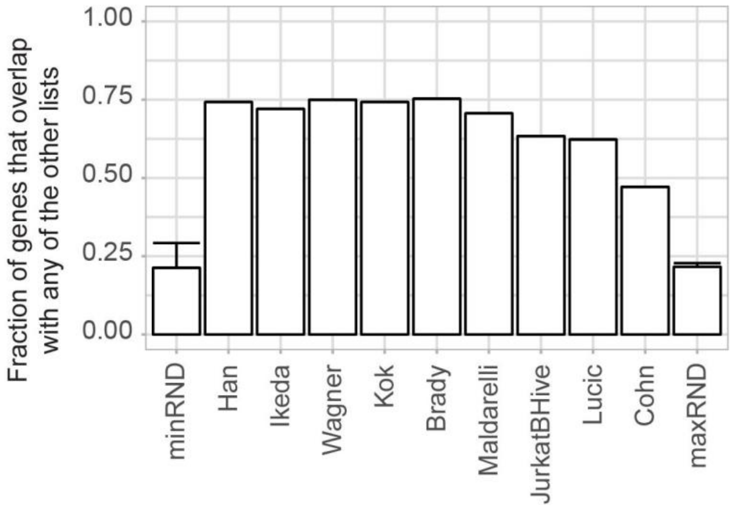

Supplementary Figure 3.
Comparison of insertion sites in $CD4^+$ T cells with B-HIVE insertion sites in Jurkat. The fraction of genes from a data set that is shared with at least one other data set is shown on the Y-axis. The bar plot shows that different data sets share most of targeted genes among each other, while randomly chosen subsets of genes (minRND and maxRND) are only partially shared with genes from other data sets.

Supplementary Figure 4

A

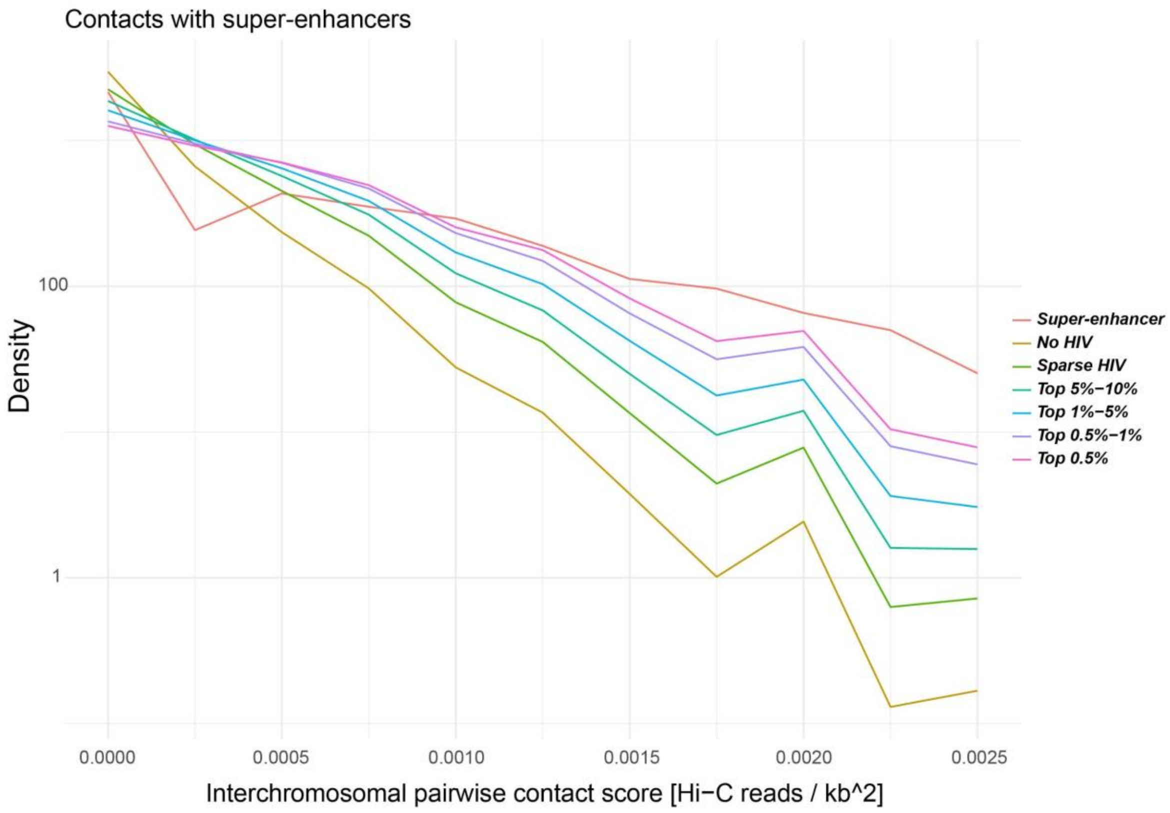

Supplementary Figure 4.
A) Density plot of inter-chromosomal pairwise contact scores (see Methods) between super-enhancers and HIV hotspots. HIV hotspots were identified within genomic bins of 100 kb, then sorted by count of HIV insertions and split in percentile groups, *e.g.*, top 0.5% in HIV-1 count, from 0.5% to 1%, *etc*. Super-enhancers show strongest inter-chromosomal contacts with other super-enhancers (observe the higher density at higher contact scores, red line), followed by contacts between super-enhancers and the most HIV-dense hotspots (pink line). The interaction scores show a monotonous decay as the HIV-1 hotspots are more sparse.

Supplementary Figure 5

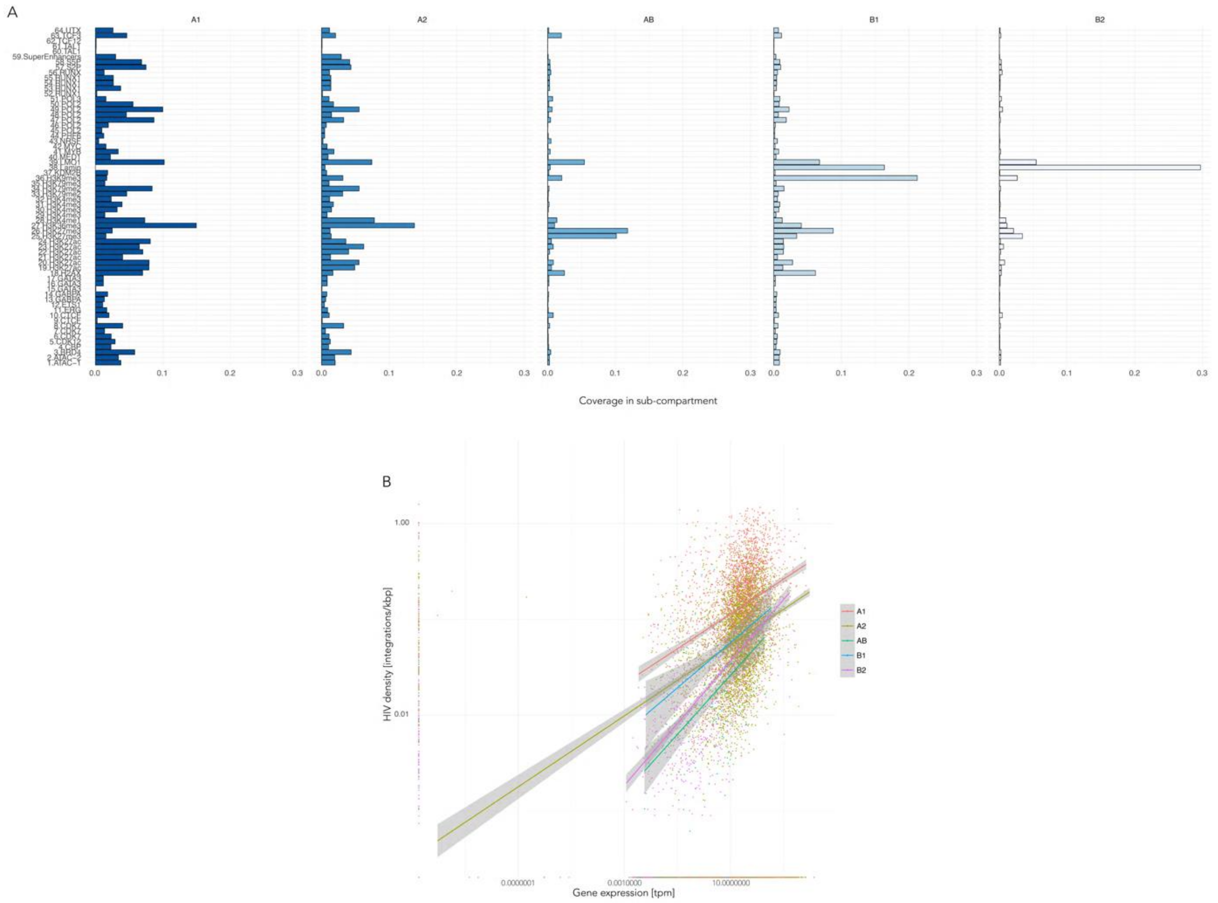

Supplementary FIgure 5.

A) Proportion of 3D sub-compartments covered by Jurkat chromatin features available from the literature. Coverage was computed as the span of enriched ChIP-Seq signal divided by the sub-compartment size.

B) Scatter plot of HIV density in gene bodies versus endogenous expression in Jurkat cells. Each dot represents a protein coding gene. Dot colors identify the 5 different sub-compartments. Sub-compartments A1 and A2 show similar distributions of gene expression and almost identical effects of gene expression on HIV-1 density (see slopes of linear models). However, A1 shows higher HIV-1 density overall compared to A2 (see vertical shift of fitted lines), suggesting an intrinsic preference of HIV-1 for A1 independent of the gene expression level.

Supplementary Figure 6

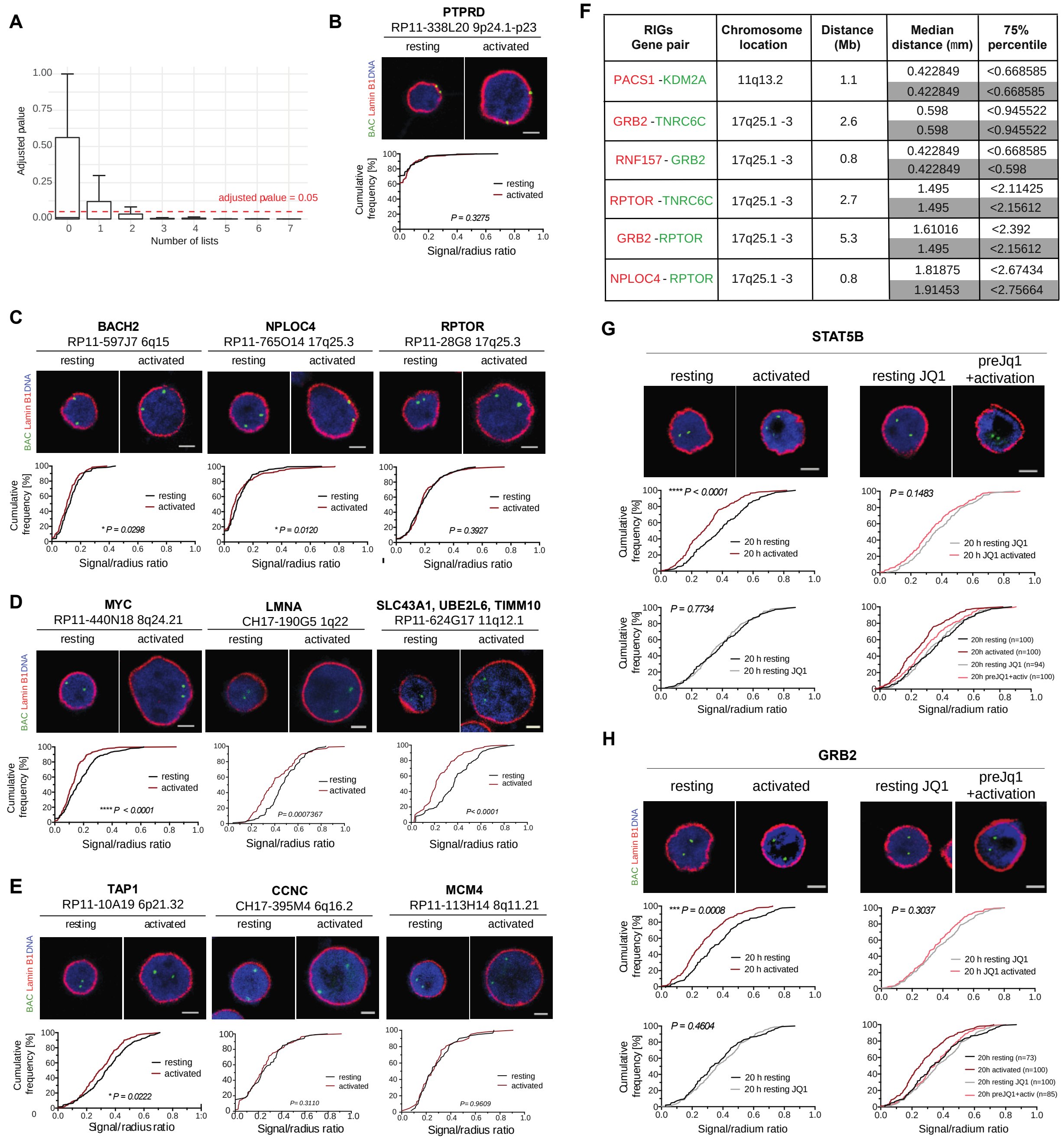

RIGs Gene pair	Chromosome location	Distance (Mb)	Median distance (㎜)	75% percentile
PACS1 -KDM2A	11q13.2	1.1	0.422849	<0.668585
			0.422849	<0.668585
GRB2 -TNRC6C	17q25.1 -3	2.6	0.598	<0.945522
			0.598	<0.945522
RNF157 - GRB2	17q25.1 -3	0.8	0.422849	<0.668585
			0.422849	<0.598
RPTOR -TNRC6C	17q25.1 -3	2.7	1.495	<2.11425
			1.495	<2.15612
GRB2 -RPTOR	17q25.1 -3	5.3	1.61016	<2.392
			1.495	<2.15612
NPLOC4 - RPTOR	17q25.1 -3	0.8	1.81875	<2.67434
			1.91453	<2.75664

Supplementary Figure 6.

A) Adjusted p-value for change in expression of genes upon activation of CD4^{+} T cells. Genes are grouped by number of HIV-1 lists they appear in. The dashed red line represents an adjusted p-value of 0.05.

3D immuno-DNA FISH in resting and activated CD4^{+} T cells. Representative images of **B)** *PTPRD.* **C)** *BACH2*, *NPLOC4* and *RPTOR*. **D)** *MYC*, *LMNA* and *SLC43A1*, *UBE2L6*, *TIMM10*. **E)** *TAP1*, *CCNC* and *MCM4.* **F)** Table summarizing the spatial relationships on chromosome 11 and 17 in resting and activated CD4^{+} T cells.

Effect of super-enhancer disruption by JQ1 on the T cell activation-induced movement of RIGs : 3D immuno-DNA FISH images of *STAT5B* **G)** and *GRB2* **H)** in $CD4^+$ T cells treated with 500 nM JQ1 or DMSO, and activated for 20 h: Green: gene, red: lamin B1, blue: DNA counterstaining with Hoechst 33342. Cumulative frequency plots in the lower panels show combined data from both experiments (n = 100, black: resting cells, red: activated cells). The p-values from the Kolmogorov-Smirnov tests are indicated.

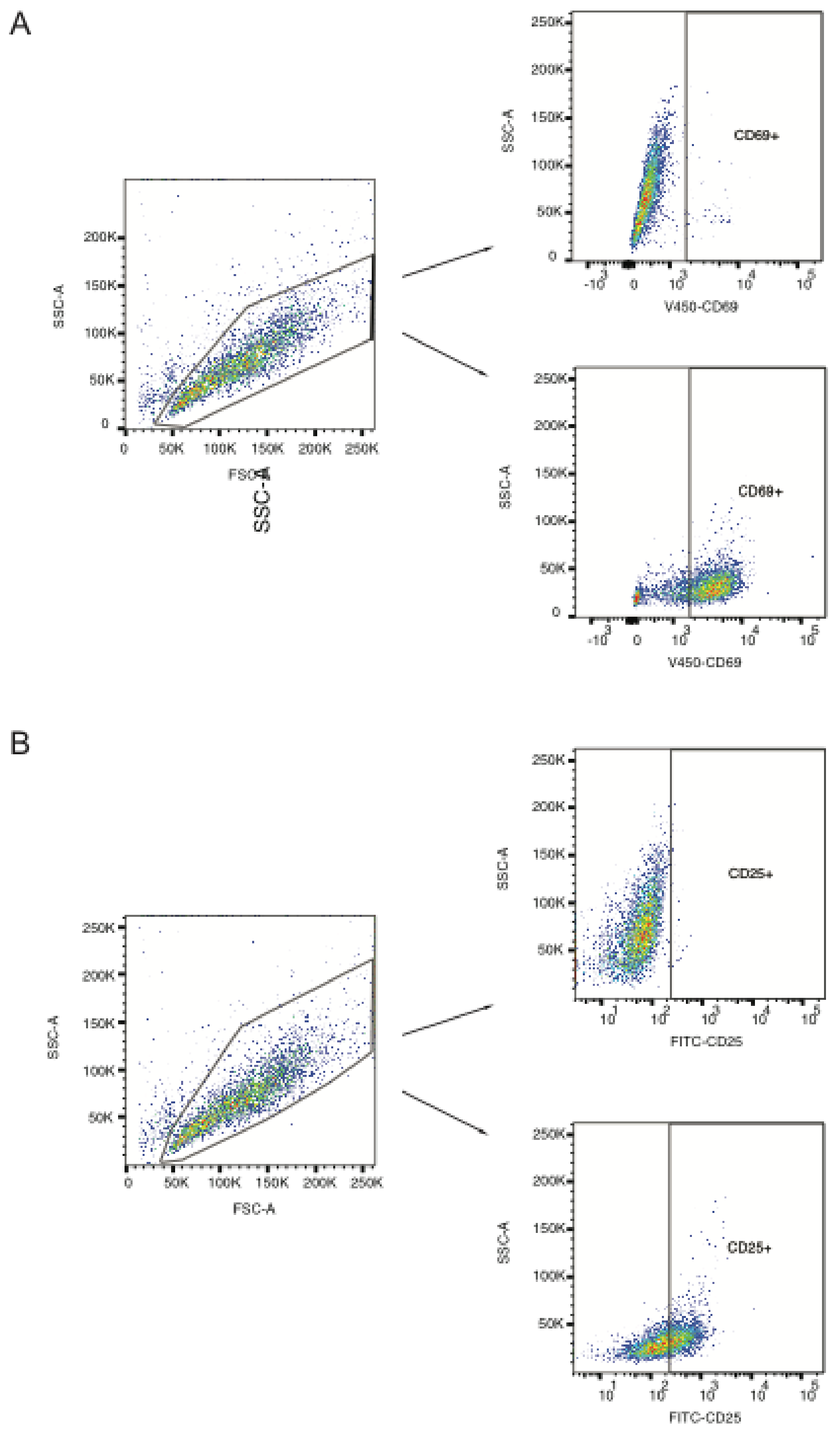

Supplementary Figure 7.
Gating strategy used to determine non activated (upper right panels) and activated cells (lower panels) with two markers, CD69(A) and CD25(B). Here shown is an example of cell activation of 20hrs, as explained in Supplementary Figure 6. Population of live unstained activated cells was used to set the gates: side scatter plus specific marker was used for activated T cell population identification.

	Lucic	Maldarelli	Cohn	Han	Ikeda	Wagner	Kok	Brady	In Vitro	Patients	Total CD4 HIV
Total number of unique IS	3167	1723	6416	74	366	443	497	864	4031	9519	13546
# of unique IS in genes	2644	1506	4644	61	320	370	444	750	3394	7345	10735
% of unique IS in genes	83	87	72	82	87	84	89	87	84	77	79
Total number of genes	57763	57763	57763	57763	57763	57763	57763	57763	57763	57763	57763
# of genes with IS	2158	1199	3012	66	305	324	445	683	2579	4253	5601
% of genes with IS	4	2	5	0	1	1	1	1	4	7	1
# of genes without IS	55605	56564	54751	57697	57458	57439	57318	57080	55184	53510	52162

Supplementary Table 1.
HIV-1 integration sites from primary CD4$^+$ T cells.
Number of unique integration sites from each list used in this study (Total number of unique IS), number and percentage of unique IS in genes (# and % of unique IS in genes), the total number of genes used in this study (Total number of genes), number and percentage of genes with integration sites used in this study (# and % of genes with IS) and number of genes without integration isets (# of genes without IS).

Primer ID	Sequence	FW or RV	Index	Description
GAT1786	5'-AATGATACGGCGACCACCGAGATCTACACTCTTTCCCTACACGACGCTCTTCCGATCTCTTTGGGAGTGAATTAGCCCTT-3'	RV	-	
GAT1787	5'-CAAGCAGAAGACGGCATACGAGATTAAATCGTGACTGGAGTTCAGACGTGTGCTCTTCCGATCTGGGCCAGGGGTCAGATAT-3'	FW	TAAATC	
GAT1788	5'-CAAGCAGAAGACGGCATACGAGATAATGCGGTGACTGGAGTTCAGACGTGTGCTCTTCCGATCTGGGCCAGGGGTCAGATAT-3'	FW	AATGCG	
GAT1789	5'-CAAGCAGAAGACGGCATACGAGATACTCGGGTGACTGGAGTTCAGACGTGTGCTCTTCCGATCTGGGCCAGGGGTCAGATAT-3'	FW	ACTCGG	
GAT1790	5'-CAAGCAGAAGACGGCATACGAGATTGTTTGGTGACTGGAGTTCAGACGTGTGCTCTTCCGATCTGGGCCAGGGGTCAGATAT-3'	FW	TGTTTG	
GAT1791	5'-CAAGCAGAAGACGGCATACGAGATGACTCCGTGACTGGAGTTCAGACGTGTGCTCTTCCGATCTGGGCCAGGGGTCAGATAT-3'	FW	GACTCC	
GAT1792	5'-CAAGCAGAAGACGGCATACGAGATAGCGGAGTGACTGGAGTTCAGACGTGTGCTCTTCCGATCTGGGCCAGGGGTCAGATAT-3'	FW	AGCGGA	Isolation HIV integration sites from 5'LTR
GAT1793	5'-CAAGCAGAAGACGGCATACGAGATTTCGTCGTGACTGGAGTTCAGACGTGTGCTCTTCCGATCTGGGCCAGGGGTCAGATAT-3'	FW	TTCGTC	
GAT1794	5'-CAAGCAGAAGACGGCATACGAGATGAAACAGTGACTGGAGTTCAGACGTGTGCTCTTCCGATCTGGGCCAGGGGTCAGATAT-3'	FW	GAAACA	
GAT1795	5'-CAAGCAGAAGACGGCATACGAGATTAACCGGTGACTGGAGTTCAGACGTGTGCTCTTCCGATCTGGGCCAGGGGTCAGATAT-3'	FW	TAACCG	
GAT1796	5'-CAAGCAGAAGACGGCATACGAGATTCTGGAGTGACTGGAGTTCAGACGTGTGCTCTTCCGATCTGGGCCAGGGGTCAGATAT-3'	FW	TCTGGA	
GAT1797	5'-CAAGCAGAAGACGGCATACGAGATAGGGACGTGACTGGAGTTCAGACGTGTGCTCTTCCGATCTGGGCCAGGGGTCAGATAT-3'	FW	AGGGAC	
GAT1798	5'-CAAGCAGAAGACGGCATACGAGATTCATTAGTGACTGGAGTTCAGACGTGTGCTCTTCCGATCTGGGCCAGGGGTCAGATAT-3'	FW	TCATTA	
GAT1799	5'-AATGATACGGCGACCACCGAGATCTACACTCTTTCCCTACACGACGCTCTTCCGATCTTAGTCAGTGTGGAAAATCTCTAG-3'	FW	-	
GAT1800	5'-CAAGCAGAAGACGGCATACGAGATTAGTTTGTGACTGGAGTTCAGACGTGTGCTCTTCCGATCTCACTACTTTGAGCACTCAAG-3'	RV	TAGTTT	
GAT1801	5'-CAAGCAGAAGACGGCATACGAGATGCTAAGGTGACTGGAGTTCAGACGTGTGCTCTTCCGATCTCACTACTTTGAGCACTCAAG-3'	RV	GCTAAG	
GAT1802	5'-CAAGCAGAAGACGGCATACGAGATGGACGAGTGACTGGAGTTCAGACGTGTGCTCTTCCGATCTCACTACTTTGAGCACTCAAG-3'	RV	GGACGA	
GAT1803	5'-CAAGCAGAAGACGGCATACGAGATCTCGAAGTGACTGGAGTTCAGACGTGTGCTCTTCCGATCTCACTACTTTGAGCACTCAAG-3'	RV	CTCGAA	
GAT1804	5'-CAAGCAGAAGACGGCATACGAGATCGTACCGTGACTGGAGTTCAGACGTGTGCTCTTCCGATCTCACTACTTTGAGCACTCAAG-3'	RV	CGTACC	
GAT1805	5'-CAAGCAGAAGACGGCATACGAGATCCTTACGTGACTGGAGTTCAGACGTGTGCTCTTCCGATCTCACTACTTTGAGCACTCAAG-3'	RV	CCTTAC	Isolation HIV integration sites from 3'LTR
GAT1806	5'-CAAGCAGAAGACGGCATACGAGATCCGTGGGTGACTGGAGTTCAGACGTGTGCTCTTCCGATCTCACTACTTTGAGCACTCAAG-3'	RV	CCGTGG	
GAT1807	5'-CAAGCAGAAGACGGCATACGAGATCGCCGGGTGACTGGAGTTCAGACGTGTGCTCTTCCGATCTCACTACTTTGAGCACTCAAG-3'	RV	CGCCGG	
GAT1808	5'-CAAGCAGAAGACGGCATACGAGATAGTTCTGTGACTGGAGTTCAGACGTGTGCTCTTCCGATCTCACTACTTTGAGCACTCAAG-3'	RV	AGTTCT	
GAT1809	5'-CAAGCAGAAGACGGCATACGAGATAGACCCGTGACTGGAGTTCAGACGTGTGCTCTTCCGATCTCACTACTTTGAGCACTCAAG-3'	RV	AGACCC	
GAT1810	5'-CAAGCAGAAGACGGCATACGAGATGGATTTGTGACTGGAGTTCAGACGTGTGCTCTTCCGATCTCACTACTTTGAGCACTCAAG-3'	RV	GGATTT	
GAT1811	5'-CAAGCAGAAGACGGCATACGAGATGGTAGTGTGACTGGAGTTCAGACGTGTGCTCTTCCGATCTCACTACTTTGAGCACTCAAG-3'	RV	GGTAGT	

Supplementary Table 2.
List of primers used in inverse PCR to map insertion sites in primary CD4$^+$ T cells. Primer ID, Sequence, FW or RW, Indexes and description.

ChIP	Source	Conducted on	Experiment	Accession number
BRD4	Hertweck A et al.		Homo sapiens Th1 RA Brd4	SRR2971477
Input	Uni W. Reference Epigenome Mapping Project	-	ChIP-Seq Input of CD4 Primary Cells	SRR787508
Input	Uni W. Reference Epigenome Mapping Project	-	ChIP-Seq Input of CD4 Primary Cells	SRR787509
Input	This study	05.2015.	ChIP-Seq Input of CD4 Primary Cells	
Input	This study	05.2015.	ChIP-Seq Input of CD4 Primary Cells	
Input	This study	08.2015.	ChIP-Seq Input of CD4 Primary Cells	
Input	This study	06.2016.	ChIP-Seq Input of CD4 Primary Cells	
Input	This study	07.2016.	ChIP-Seq Input of CD4 Primary Cells	
H3K27ac	This study	07.2016.	Histone H3K27ac ChIP-Seq of CD4 Primary Cells	
H3K27ac	This study	07.2016.	Histone H3K27ac ChIP-Seq of CD4 Primary Cells	
H3K27ac	This study	08.2015.	Histone H3K27ac ChIP-Seq of CD4 Primary Cells	
H3K27ac	This study	08.2015.	Histone H3K27ac ChIP-Seq of CD4 Primary Cells	
H3K36me3	This study	07.2016.	Histone H3K27ac ChIP-Seq of CD4 Primary Cells	
H3K36me3	This study	07.2016.	Histone H3K27ac ChIP-Seq of CD4 Primary Cells	
H3K27me3	Uni W. Reference Epigenome Mapping Project	-	Histone H3K27me3 ChIP-Seq of CD4 Primary Cells	SRR980445
H3K27me3	Uni W. Reference Epigenome Mapping Project	-	Histone H3K27me3 ChIP-Seq of CD4 Primary Cells	SRR980446
H3K4me1	Uni W. Reference Epigenome Mapping Project		Histone H3K4me1 ChIP-Seq of CD4 Primary Cells	SRR980417
H3K4me1	Uni W. Reference Epigenome Mapping Project		Histone H3K4me1 ChIP-Seq of CD4 Primary Cells	SRR980418
H3K4me3	This study	05.2015.	Histone H3K4me3 ChIP-Seq of CD4 Primary Cells	
H3K4me3	This study	05.2015.	Histone H3K4me3 ChIP-Seq of CD4 Primary Cells	
H3K4me3	This study	08.2015.	Histone H3K4me3 ChIP-Seq of CD4 Primary Cells	
H3K9me2	This study	05.2015.	Histone H3K9me2 ChIP-Seq of CD4 Primary Cells	
H3K9me2	This study	08.2015.	Histone H3K9me2 ChIP-Seq of CD4 Primary Cells	
H3K9me2	This study	08.2015.	Histone H3K9me2 ChIP-Seq of CD4 Primary Cells	
H4K20me1	This study	08.2015.	Histone H4K20me1 ChIP-Seq of CD4 Primary Cells	
H4K20me1	This study	08.2015.	Histone H4K20me1 ChIP-Seq of CD4 Primary Cells	
MED1	Hertweck A et al.	-	Homo sapiens Th1 RA Med1	SRR2971479

Supplementary Table 3.
List of ChIP-Seq data.
All immuno-precipitated factors, source, the date experiment was performed, the name of the experiment and accession numbers.

SAMPLE	Concentration(ng/ul)	Volume(ul)	Sample_Information
1a	56	14	donor 41 CD4T cells CTRL resting
1b	56	14	donor 41 CD4T cells CTRL resting
2a	70	14	donor 41 CD4T cells JQ1 resting
2b	70	14	donor 41 CD4T cells JQ1 resting
3a	95	12	donor 42 CD4T cells CTRL resting
3b	95	12	donor 42 CD4T cells CTRL resting
4a	82	12	donor 42 CD4T cells JQ1 resting
4b	82	12	donor 42 CD4T cells JQ1 resting
5a	111	12	donor 44 CD4T cells CTRL resting
5b	111	12	donor 44 CD4T cells CTRL resting
6a	81	12	donor 44 CD4T cells JQ1 resting
6b	81	12	donor 44 CD4T cells JQ1 resting
7a	842	10	donor 41 CD4T cells CTRL activated
7b	842	10	donor 41 CD4T cells CTRL activated
8a	576	10	donor 41 CD4T cells JQ1 activated
8b	576	10	donor 41 CD4T cells JQ1 activated
9a	522	10	donor 42 CD4T cells CTRL activated
9b	522	10	donor 42 CD4T cells CTRL activated
10a	470	10	donor 42 CD4T cells JQ1 activated
10b	470	10	donor 42 CD4T cells JQ1 activated
11a	882	10	donor 44 CD4T cells CTRL activated
11b	882	10	donor 44 CD4T cells CTRL activated
12a	684	10	donor 44 CD4T cells JQ1 activated
12b	684	10	donor 44 CD4T cells JQ1 activated

Supplementary Table 4
List of RNA-Seq data.
Sample number, sample concentrations, volume and sample ID.

Covered gene	BAC clone	Posi1on GRCh37	Chr bands	Posi1on GRCh38
BACH2	RP11*597J7	chr6:90,681,035*90,881,332	q15	chr6:89,971,316*90,171,613
FOXP1	RP11*905F6	chr3:71,256,411*71,475,547	p13	chr3:71,207,260*71,426,396
GRB2	RP11*16C1	chr17:73,269,580*73,422,789	q25.1	chr17:75,273,499*75,426,708
KDM2A	RP11*157K17	chr11:66,913,936*67,086,969	q13.2	chr11:67,146,465*67,319,498
MKL2	RP11*1072B15	chr16:14,245,800*14,422,293	p13.12	chr16:14,151,943*14,328,436
MYC	RP11*440N18	chr8:128,596,756*128,777,986	q24.21	chr8:127,584,511*127,765,740
NFATC3	RP11*67A1	chr16:68,111,243*68,156,174	q22.1	chr16:68,077,340*68,122,271
NPLOC4	RP11*765O14	chr17:79,379,432*79,579,283	q25.3	chr17:81,405,632*81,612,257
PACS1	RP11*675B4	chr11:65,815,062*65,953,271	q13.1*q13.2	chr11:66,047,591*66,185,800
PTPRD	RP11*338L20	chr9:8,981,678*9,142,717	p24.1*p23	chr9:8,981,678*9,142,717
RNF157	RP11*449J21	chr17:73,999,159*74,183,053	q25.1	chr17:76,003,078*76,186,972
RPTOR	RP11*28G8	chr17:78,705,399*78,868,353	q25.3	chr17:80,731,599*80,894,553
STAT5B	CTD*3124P7	chr17:40,326,868*40,479,760	q21.2	chr17:42,174,850*42,327,742
TAP1	RP11*10A19	chr6:32,735,717*32,915,875	p21.32	chr6:32,767,940*32,948,098
TNRC6C	RP11*153A23	chr17:76,004,952*76,182,689	q25.3	chr17:78,008,871*78,186608
LMNA	CH17*190G5	chr1:156,020,485*156,242,095	1q22	chr1:156,050,694*156,272,304
SLC43A1, UBE2L6, TIMM10	RP11*624G17	chr11:57,196,976*57,407,534	11q12.1	chr11:57,429,503*57,640,061
CCNC	CH17*395M4	chr6:99,928,076*100,138,693	6q16.2	chr6:99,480,200*99,690,817
MCM4	RP11*113H14	chr8:48,815,695*48,977,857	8q11.21	chr8:47,903,135*48,065,297
MSN	CH17*413H2	chrX:64,779,881*64,988,569	Xq12	chrX:65,560,001*65,768,727
RECQL	RP11*501E24	chr12:21,521,462*21,700,905	12p12.1	chr12:21,368,528*21,547,971
MARCH1	CH17*454P4	chr4:164,780,476*164,991,773	4q32.3	chr4:163,859,324*164,070,621

Supplementary Table 5 List of BACs.
Gene covered by bacterial artificial chromosomes used in 3D immuno-DNA FISH experiments (Covered gene), clone identification (BAC clone) and BACs genomic coordinates (Position GRCh37, Chr bands and Position GRCh38).

Supplementary methods

Linear amplification-mediated PCR (LAM-PCR) to map HIV-1 insertion sites in primary cells

The mapping was performed as described previously[1,2] using 1µg of genomic DNA from HIV-1 NL4-3 infected primary human $CD4^+$ T cells.

Inverse PCR to map HIV-1 insertion sites in primary cells

The mapping of HIV was performed based on the protocol published by Chen *et al.* [3] with modifications. Briefly, 3 µg genomic DNA from HIV-1NL4_3-infected $CD4^+$ T cells treated with 500 nM JQ1 or DMSO before infection were digested by 2 µL 10,000 U/mL AluI (NEB, R0137S) and 2 µL 10,000 U/mL BglII (NEB, R0144S) in NEBuffer 2.1 in 50 µL final volume at 37 ℃ for 3 hours. The reaction was heat-inactivated at 80 ℃ for 20 min. BglII digestion aims to eliminate byproducts, which contain only the sequence of the HIV-1 backbone after AluI digestion. The double-digested products were diluted in 1 mL T4 DNA ligase buffer, then self-ligated by adding 2 µL 30 U/µL T4 DNA ligase (Thermo Fisher Scientific, EL0013) and incubating at 16 ℃ overnight. The ligation reaction was ethanol-precipitated the following day. The pellet was resuspended in 84 µL distilled water. To destroy non-circularized genomic DNA, 4 µL 25 mM ATP and 2 µL 10 U/µL Plasmid-Safe™ ATP-Dependent DNase (Epicentre, E3101K) were added with 10X Reaction Buffer in 100 µL final volume at 37 ℃ for 2 hours. The reaction was heat-inactivated at 70 ℃ for 30 min.

6 µL Plasmid-Safe-digested products were mixed in 50 µL standard Phusion polymerase reaction mix (Thermo Fisher Scientific, F530S) in GC buffer, with 0.1 µM primers GAT1786 (annealing to the Illumina PE1.0 primer) and one indexing primer GAT-int_5LTR (annealing to the 5' end of the LTR) or 0.1 µM primers GAT1799 (annealing to the Illumina PE1.0 primer) and one indexing primer GAT-int_3LTR (annealing to the 3' end of the LTR) for each condition of the sample. The cycling conditions were as follows: 98 ℃ for 1 min; 98 ℃ for 20 sec, 55 ℃ for 1 min, 72 ℃ for 5 min (2 cycles); 98 ℃ for 20 sec, 62 ℃ for 1 min, 72 ℃ for 5 min (27 cycles); 72 ℃ for 5 min. GAT-int_5LTR and GAT-int_3LTR primers add the Illumina PE2.0 primer and a 6-nucleotide index to the amplicons. PCR products ran as a smear on agarose gel . The primers used are described in **Supplementary Table 2**.

Integration sites and genes

We analyzed eight lists of HIV-1 integration sites. [[4–9]] were downloaded from retroviral integration database (RID)[10]. We downloaded raw sequences from [[11]] (PRJNA531196) and one data set was provided by Lusic lab (previously unpublished).

We processed the raw sequences from [11] in the following way: To remove barcodes + LTR-CA sequence on the left side of the reads and linker sequence on the right, we used bbduk allowing for editdistance=2 and minlength=0. Next, reads were converted to fasta files and mapped to hg19 (GRCh37) by pBLAT with parameters -maxIntron=0 -minIdentity=98. We processed the resulting tables in R in the following way: we calculated the percentage of identity by dividing the number of matches by query length for each sequence. All alignments with percentage of identity calculated in this way smaller than 98.0 were not considered. All sequences which mapped to multiple positions in the genome were also not further considered. In this way we obtained in total 1475 uniquely mappable integration sites in activated cells. Some of those integration sites originated from clonally expanded cells, and those were collapsed for our analysis. We obtained 864 unique integration sites from activated cells. Code for processing raw sequences to get integration sites is available here: https://github.com/gui11aume/genome_structure_and_HIV_integration/blob/master/maja/Brady_Integration_Sites.md .

We used only unique integrations from each study. If the location of the integration was not precisely defined (spanning more than one nucleotide), we used the midpoint as the location for that integration. All sites were converted to hg19 (GRCh37) version of the genome using R rtracklayer package[12]. Gene coordinates were downloaded from Ensembl, GRCh37, February 2014. UCSC symbols were used for genes in UCSC (hg19), while others were named after ENSG identifier. Integration sites used in this analysis are available as an R object containing a list of GRanges objects, one list element for each data set used, https://github.com/gui11aume/genome_structure_and_HIV_integration/blob/master/maja/is.Robj.

In those cases where using all genes could have introduced bias to the results (for ChIP-Seq profiles on genes and expression data), only protein coding genes were used for the analysis. This was the case in the following figures: Figure 1A, Figure 2A, 2B, 2C, 2D, Figure 4F, Supplementary Figure 2 (E, G) and Supplementary Figure 5B. List of all genes from Ensembl, GRCh37 assembly from February 2014 was used for the Figures 1C, Supplementary Figure 1 (A, B, C, D) and Supplementary Figure 3.

We counted overlaps between integration sites and all GRCh37 genes disregarding strand and orientation.

Gene coordinates for genes and number of HIV integration lists can be found in **Supplementary Data 1 .**

To control for the JQ1 activity we then additionally sequenced 39k (14k of IS in non-treated ie control infections and 25k in JQ1 pretreated cells).

All data on primary cells (i.e. a total of ~28k) were then used for comparison with insertion sites in Jurkats.

Redefinition of RIGs

To each gene, we added a number representing number of lists that found HIV-1 integration inside this gene (**Supplementary Data 1**). We define recurrent integration genes (RIGs) as genes for which we found HIV integration in 2 or more data sets. To account for possible false positives, we assign an assessment of confidence to all RIGs we defined by doing the following: for each of the 8 mapping experiments, we produced 100 mock data sets with the same number of integration sites. Random mock HIV integration sites were chosen to match the distance to the nearest expressed gene, as explained for the ROC analysis. In this way we created 100 matched control lists of 8 “experiments”. For each gene, we counted the number of control “experiments” where given gene was targeted. This value ranges from 0 (if the gene was not targeted) to 8 (if the gene was targeted in all 8 random data sets). For each gene we collected those scores for the 100 randomizations and counted how often they were higher than the observed score. The matrix that contains number of lists each gene is found on in all 100 randomizations can be found here (in RDS format): https://github.com/gui11aume/genome_structure_and_HIV_integration/raw/master/maja/Replicates.RDS.

For each gene we assigned a number which represents the number of randomizations (out of 100) in which this gene scored worse than in real data sets (**Supplementary Data 1**, last column nRealBetter).

To assess the relationship between number of integration sites included in analysis and number of genes discovered to have integrations, we did the following: We made lists of genes found to have integrations in each study. Next, we sorted those lists in decreasing order, by number of integration sites found in a study. We plotted cumulative sum of number of integrations found in studies on x-axis, and number of genes targeted in that study and not in any other studies before that study on y axis. We used linear regression to model this relationship.

ChIP-Seq data analysis

We analysed ChIP-Seq data sets obtained from this study (H3K4me3, H3K36me3, H3K27Ac, H4K20me1 and H3K9me2) and publicly available data sets from University of Washington Human Reference Epigenome Mapping Project H3K4me1 (SRA accession number: SRX342315), H3K27me3 (SRX342313), input for CD4 primary T cells (SRX252742). Data sets for BRD4 (SRR2971477), MED1 (SRR2971478) and corresponding Input (GSM1527712) were downloaded from [13].ChIP-Seq reads were mapped to human genome (GRCh37) using Bbmap([14]) with parameters minid=0.98, qtrim=lr, minavgquality=20. Resulting bam files belonging to same experiments were merged and sorted using bamtools. Average binding profiles in reads per million across sets of genes were made using ngsplot [15]. Peaks were called using MACS2 [16], for every data set versus its matching input, with parameters --broad --broad-cutoff 0.1 -p 1e-9 -g 2.7e9 -B. All results were transformed to RPKM for downstream analysis and visualization. The list of ChIP-Seq data used in this study are in **Supplementary Table 3.**

For Jurkat cells, ChIP-Seq reads were mapped to hg19 using BWA-mem. BWA options were as follows: '-k17 -r1.3 -B2 -O4 -T22' for read lengths less or equal to 30 nt, '-k18 -B3 -O5 -T28' for read lengths less or equal to 40 nt and default options for longer reads. ChIP-Seq enriched regions were discretized using Zerone [17] with mapping quality cutoff 20 and enrichment confidence 0.99. We used publicly-available ChIP-Seq profiles for Jurkat cell line. All data sets were obtained from NCBI Gene Expression Omnibus with the following series accessions: ERG and GABPA (GSE49091) [18]; H3K27Ac, CDK7 and PolII (GSE50622, GSE60027) [19]; H3K36me3, H3K79me3, H3K4me1, H3K9me3, H3K27Ac, H3K4me3, PolII, S5P and S2P (GSE65687) [20]; NRSF (GSE53366) [21]; PolII and CDK12 (GSE72023) [22]; ETS1, CBP and RUNX (GSE17954) [23]; H3K27Ac (GSE51522) [24]; PolIII (GSE20309) [25]; H3K27Ac and H3K27me3 (GSE59257) [26]; H3K4me3, H3K27me3, H3K79me2 and PolII (GSE23080); PHF6 (GSE45864); KDM2B (GSE70624) [27]; RUNX1, GATA3, TAL1, LMO1, TCF3 and TCF12 (GSE29181) [28]**;** H3K27Ac, MED1 and MYB (GSE59657) [29]; H3K4me3 (GSE35583) [30]; Lamin (DamID, GSE94971) [31]; RUNX1 (GSE42575) [32]; TAL1 (GSE25000) [33]; H3K4me3 and H3K79me2 (GSE60104) [34]; PolII (GSE25494) [35]; RUNX1, GATA3, H3K27Ac and CTCF (GSE68976) [36]; MYC, BRD4 and CDK7 (GSE83777); RUNX1 and GATA3 (GSE76181) [37]; CTCF (GSE12889) [38]; H2AX (GSE25577) [39]; UTX (GSE72300) [33]; YY1 (GSE99521) [40].

Super-enhancer calling

We used super-enhancer data for all activated $CD4^+$ cell types CD4p_CD25-_Il17p_PMAstim_Th17 and CD4p_CD25-_Il17-_PMAstim_Th from dbSuper[41]. To define super-enhancers using our own ChIP-Seq data we followed the same procedures as in dbSuper. Thus, we defined super-enhancers using HOMER software [41,42] findPeaks with default parameters ('-style super') on our H3K27ac peaks (peak finding described above). Briefly, peaks found within a distance of 12.5 kb were stitched together into larger regions. Super-enhancer signal of each region was determined by the total normalized number of reads subtracted by normalized number of reads in the input peaks. Regions are sorted by score and super-enhancers are identified as regions with score higher than that defined by slope greater than 1. We defined a gene to be “proximal” to super-enhancer if it overlaps with one, or if we can find a super-enhancer element 5 kb upstream of transcription start site.

Hi-C contacts

Hi-C reads were mapped using BWA-MEM with the following options: ‘-P -k17 -U0 -L0,0 -T25’. Each read end was mapped independently. Genuine Hi-C contacts were validated with the Hi.C pipeline (https://github.com/ezorita/hi.c), using the following discard filters: (i) contact pairs with mapping quality below 10, (ii) self-circularized molecules and (iii) reads with inferred insert size

greater than 2000 bp after digestion and ligation. Hi-C contacts were then binned at 5kb resolution and stored in HDF5 format using Cooler (https://github.com/mirnylab/cooler).

Hotspots and HIV-dense genes

HIV genome-wide hotspots were identified dividing the genome in bins of 100 kb and sorting the bins by HIV insertion count. HIV density in genes followed a similar rationale but the bins were designed to match gene bodies, as described by ENSEMBL GTF GRCh37 release 75. HIV density was computed as the number of insertions per kb of gene body.

AB score

AB scores were derived from the first eigenvector of the Hi-C correlation matrix (as in Identification of 3D sub-compartments). We chose the reference A and B regions to be the most dissimilar 3D structures, i.e. the genomic bins with 10% top and bottom values of the first eigenvector, respectively. For each row of the correlation matrix we computed A_{score} and B_{score} as the sum of its values in the A and B reference regions, respectively. Finally, the AB score was computed as:

$$AB_{score} = \frac{A_{score} - B_{score}}{A_{score} + B_{score}} \cdot 100$$

Yielding values between 100 for A-like regions and -100 for B-like regions. Ambiguous regions that are equally in contact with the reference A and B regions, or that are not in contact with them at all, will have AB scores close to 0.

Identification of 3D sub-compartments

The following pre-processing steps were performed to prepare the matrix before clustering. Observed-over-expected normalization was applied to smooth the diagonal decay[43], followed by ICE row-sum balancing[44]. Outlier contacts, such as enhancer-promoter loops, were smoothed by thresholding the largest values of the matrix to the 90-th percentile. The correlation matrix was subsequently computed and the resulting diagonal was set to 0. The outliers of the correlation matrix were further smoothed by applying a linear scaling, *i.e.* values below the 5-th and above the 95-th percentiles were set to -1 and +1, respectively, and intermediate values were scaled proportionally. Spatial clusters were identified on the correlation matrix running k-means (10 restarts) with the first *k*=15 weighted eigenvectors, *i.e.* the 15 leading eigenvectors, each weighted by its respective eigenvalue. This process was repeated independently for each chromosome, delineating 15 spatial clusters per chromosome.

The choice of *k* relied on previous Hi-C analyses, which reported six distinguishable clusters[45]. Comparatively, the present clustering is performed at higher resolution (5kb). For this reason, the value of *k* was chosen large enough to allocate new potentially unresolved conformations. The enrichment of ChIP-Seq data on the intrachromosomal clusters showed, in most cases, five clear patterns (supplementary figure with ChIP heatmap, intrachromosomal). Therefore, *k* was reduced to 5 in the subsequent interchromosomal clustering. The same analysis was repeated with increasing values of *k*, resulting in split subclusters which shared similar features.

To identify interchromosomal clusters, normalized interchromosomal scores were computed between each pair of chromosomal sub-compartments. Normalized scores were defined as the

total number of Hi-C reads between them, divided by the product of their sizes. The final sub-compartments were identified by k-means clustering on the normalized score matrix with the *k*=5 leading weighted eigenvectors. Compartment names A1, A2, AB, B1 and B2 were assigned based on their distribution on the AB score scale (Figure 5C). A1 and A2 had strong and moderate enrichment in active transcription marks, respectively. AB, B1 and B2 showed very low levels of active marks and moderate to strong enrichment in H3K27me3, H3K9me3 and Lamin, respectively (Supplementary figure with interchromosomal heatmap). The complete list of sub-compartments is available in the supplementary material.

The A1, A2 and B2 sub-compartments are robust to the implementation details of the definition: when we used different normalizations or different weights for the eigenvectors, they always appeared with similar coverage and chromatin features. On the other hand, AB and B1 varied in coverage and composition, suggesting that they are fuzzier than the other sub-compartments.

Pairwise contact score
Throughout the study, pairwise contact scores were used to quantify the amount of 3D interactions between multiple loci on different chromosomes. Intrachromosomal contacts were not considered in this computation in order to avoid the intrinsic bias produced by short-range 1D interactions (loci that are closer in 1D tend to interact more in 3D). Pairwise scores were computed as the sum of Hi-C contacts within the interchromosomal region covered by a pair of loci, divided by the product of their lengths (in kbp). The unit of this metric (number Hi-C reads per square kilobase pair) allows for fair comparison of different loci even if their spans are different.

ROC analysis
In order to assess if there is an enrichment of various chromatin features on sites of HIV-1 integration, we adapted the ROC curve areas method from [46] and [47]). In short, the strategy was to use “nested case controls” - a collection of integration sites sampled from the genome which would act as control sites and can be compared to true integration sites. For every chromatin feature and experiment we analysed, we compared density of values for this feature measured on integration sites, versus density of values of this feature measured on control sites. For every cut-point of value of measured feature, we measured the percentage of integration sites with value of this feature higher than the cutpoint (true positive rate) and percentage of control sites with value of this feature higher then the cut-point (false positive rate). Thus, we constructed the ROC curve by calculating the true and false positive rates for all possible cut-point values for analysed epigenomic feature. The area under the ROC curve was then calculated. For details, see supplementary Text S1 [47]). The control sites were generated to account for bias of integration towards genes - they were sampled to match true sites in distance to nearest gene. For each true integration site, we generated 10 matched control sites and compared various chromatin features of the matched sites with the chromatin features of the true site. For each true integration site and chromatin feature, we counted a fraction of control sites having a lower feature value (e.g. true site has higher H3K27ac value than n percent of control matched sites). We averaged the results over all experiments. This is explained in more detail in the following sections:

For random matched control sites,e generated 10 control sites for every integration site in the following way: First we generated random 100 million numbers from 1 to largest chromosome length with seed set to 23779. Next, we generated 100 million chromosome names, where names were chosen at random but with weights corresponding to the number of occurrences of each chromosome in our data set. This way we generated 100 million random possible positions for controls. Next, we excluded all the positions from this random set that were found in the blacklisted area of human GRCh37 genome [48]. Sites are available on request. We calculated the distance to the nearest gene for each possible integration site and divided them into subsets of 1000 base pair bins based on those distances. For each true integration site, we extracted a subset of all random possible positions that are located in the same bin of distance to their nearest gene as the integration site is to its nearest bin. Then, from those equidistant subset of random integration sites we randomly picked 10 to represent random matched controls for each real integration site.
To Add genomic feature value to integration sites and compare integration sites to its random matched controls we firstcut the genome into tiles of length 1000 base pairs and excluded blacklisted areas. We calculated 75th quantile of RPKM values of each genomic feature (for each chromatin mark and transcription factor separately) over each tile. For super-enhancers we used 1 if super-enhancer exists in a tile, and a 0 if it does not instead of RPKM values. We assigned the value of the bin in which integration site is located to each integration site (and each random matched control). Next, we compared the value for each integration site only with its matched controls to determine the proportions of controls whose values equaled or exceeded that of the integration site. We scored each integration site in the following way: if this value was higher on true integration site than on matched control, we counted it as 1. If the value for the matched control was equal to true value on integration site, we counted it as ½, and if the value was lower, it was counted as 0. Final score for each integration site was calculated as average of those 10 values. Finally, we calculated empirical ROC area under the curve as average of all values for integrations in a data set. At last, we repeated this analysis on various bin sizes; 1Kb, 2Kb, 5Kb, 10Kb, 20Kb, 25Kb, 50Kb, 100Kb and on all genomic features and data sets and created a heatmap for every bin size (data not shown). We implemented p value calculation from [47] in R. Briefly, all comparisons utilize the Wald-test statistic and are referred to a Chi Square distribution to obtain p-values.

RNA-Seq data analysis
We mapped reads from RNA-Seq experiments to human genome (GRCh37 assembly, GENCODEV19) using BBMap with parameters maxindel=200000 xstag=unstranded ambiguous=random xmtag=t scoretag=t pairedonly=t minavgquality=20 maqb=51 minid=0.91. To calculate mean expression over replicates, we used rlog transformation from DESeq2 package [49] to normalize the counts over all replicates, and calculated mean over all replicates. We used a regularized logarithm transformation (rlog) to make our data more homoskedastic. The rlog transformation produces log2 scale transformed data which has been normalized with respect to library size. We used rlog instead of log2 transformation because it is more robust in the case when the size factors vary widely; this transformation is reducing the variance to avoid that the result becomes dominated by highly expressed, highly variable genes (original count scale data), or low expressed genes (if logarithm-transformed data are used). We used a widely utilised rlog approach of DESeq2, which enables transformation similar to a log2 for genes with high counts, and resolves the problem for genes with low counts by compressing together the values for different samples. Otherwise, a standard logarithm transformation would spread apart the data, ie random noise could overtake the real biological signal. Differential expression for JQ1 treated cells VS control cells was done with the same package, following the Bioconductor RNA-seq workflow [50]. Genes were divided to expression groups as follows: All genes with rlog of expression (averaged over replicates in activated non treated CD4+ T cells) lower or equal to

0 are considered to be not expressed. We divided the rest into 3 groups: low 10% being all genes with expression in the bottom 10% from genes that were considered as expressed. Analogously, we grouped all most highly expressed genes (top 10 quantile) into top 10% group, while the rest of the genes was grouped in mid group.

For Jurkat cells, gene expression levels were derived from mRNA-seq experiments in Jurkat . Sequencing reads were mapped to protein-coding Ensembl cDNA assembly GRCh37 release 75 using kallisto [51] with options '–single' (single-end mode), '–bias' (sequence bias correction, '–s300' (fragment length 300 nucleotides) and '–l100' (s.d. 100 nucleotides). The counts of the different isoforms were summed to make a total count per gene copy in transcripts per million reads (tpm).
The list of RNA-Seq data used in this study are in **Supplementary Table 4.**

Logistic regression
We used logistic regression to model the HIV-1 insertion landscape in Jurkat cells, based on the following four predictors: gene expression, distance of the gene to the closest super enhancer, sub-compartment of the gene and gene size. Gene size must be added to the model because it is a confounding factor. In model I, we predicted whether the gene was a typical HIV target, defined as belonging to the top 33% genes with highest HIV insertion rate (number of insertions divided by gene size). In a model II, we predicted whether the gene contained a hotspot, defined as a 10 kb bin with more than five HIV insertions (this corresponds to the top 2.5% genes with highest number of insertions in a single 10 kb bin).

The models were trained with the standard parameters of the glm function in R. We used 5-fold cross validation to test combinations of transforms (hyperbolic arcsine and logarithmic functions) and / or discretization in quantiles. The best cross-validation scores were obtained after discretizing the predictors in combinations of tertiles and quartiles, so models I and II were trained on discretized variables (the same discretization was used for both models). We then removed one of the four variables, retrained the model in the same conditions and measured the probability that a gene is classified as HIV-1 target (typical targets in model I, hotspots in model II) given that it is indeed an HIV-1 target. We chose this score instead of the classification accuracy because of the low amount of HIV-1 targets in model II – where the classification accuracy ranges from 97.5% to 100%. We used the loss of this score compared to the full model as an indicator of the intrinsic value of the predictor. The results did not change substantially when typical HIV targets were defined as top 50% or top 20%, neither when hotpots were defined as top 5% or top 1% (not shown).

Supplementary References

1. Bartholomae, C. C., Glimm, H., von Kalle, C. & Schmidt, M. Insertion Site Pattern: Global Approach by Linear Amplification-Mediated PCR and Mass Sequencing. in *Methods in Molecular Biology* 255–265 (2012).
2. Schmidt, M. *et al.* High-resolution insertion-site analysis by linear amplification-mediated PCR (LAM-PCR). *Nat. Methods* **4**, 1051–1057 (2007).
3. Chen, H. C., Zorita, E. & Filion, G. J. Using Barcoded HIV Ensembles (B-HIVE) for single provirus transcriptomics. *Curr. Protoc. Mol. Biol.* (2018).
4. Han, Y. *et al.* Resting CD4+ T cells from human immunodeficiency virus type 1 (HIV-1)-infected individuals carry integrated HIV-1 genomes within actively transcribed host genes. *J. Virol.* **78**, 6122–6133 (2004).
5. Ikeda, T., Shibata, J., Yoshimura, K., Koito, A. & Matsushita, S. Recurrent HIV-1 integration at the BACH2 locus in resting CD4+ T cell populations during effective highly active antiretroviral therapy. *J. Infect. Dis.* **195**, 716–725 (2007).
6. Maldarelli, F. *et al.* HIV latency. Specific HIV integration sites are linked to clonal expansion and persistence of infected cells. *Science* **345**, 179–183 (2014).
7. Wagner, T. A. *et al.* HIV latency. Proliferation of cells with HIV integrated into cancer genes contributes to persistent infection. *Science* **345**, 570–573 (2014).
8. Cohn, L. B. *et al.* HIV-1 integration landscape during latent and active infection. *Cell* **160**, 420–432 (2015).
9. Kok, Y. L. *et al.* Monocyte-derived macrophages exhibit distinct and more restricted HIV-1 integration site repertoire than CD4(+) T cells. *Sci. Rep.* **6**, 24157 (2016).
10. Shao, W. *et al.* Retrovirus Integration Database (RID): a public database for retroviral insertion sites into host genomes. *Retrovirology* **13**, 47 (2016).
11. Brady, T. *et al.* HIV integration site distributions in resting and activated CD4+ T cells infected in culture. *AIDS* **23**, 1461–1471 (2009).
12. Lawrence, M., Gentleman, R. & Carey, V. rtracklayer: an R package for interfacing with genome browsers. *Bioinformatics* **25**, 1841–1842 (2009).
13. Hertweck, A. *et al.* T-bet Activates Th1 Genes through Mediator and the Super Elongation Complex. *Cell Rep.* **15**, 2756–2770 (2016).
14. *BBMap: A Fast, Accurate, Splice-Aware Aligner*. (2014).
15. Shen, L., Shao, N., Liu, X. & Nestler, E. ngs.plot: Quick mining and visualization of next-generation sequencing data by integrating genomic databases. *BMC Genomics* **15**, 284 (2014).
16. Zhang, Y. *et al.* Model-based analysis of ChIP-Seq (MACS). *Genome Biol.* **9**, R137 (2008).
17. Cuscó, P. & Filion, G. J. Zerone: a ChIP-seq discretizer for multiple replicates with built-in quality control. *Bioinformatics* **32**, 2896–2902 (2016).
18. Sharma, N. L. *et al.* The ETS family member GABPα modulates androgen receptor signalling and mediates an aggressive phenotype in prostate cancer. *Nucleic Acids Res.* **42**, 6256–6269 (2014).
19. Kwiatkowski, N. *et al.* Targeting transcription regulation in cancer with a covalent CDK7

inhibitor. *Nature* **511**, 616–620 (2014).

20. Reeder, J. E., Kwak, Y.-T., McNamara, R. P., Forst, C. V. & D'Orso, I. HIV Tat controls RNA Polymerase II and the epigenetic landscape to transcriptionally reprogram target immune cells. *Elife* **4**, (2015).
21. Gasper, W. C. *et al.* Fully automated high-throughput chromatin immunoprecipitation for ChIP-seq: identifying ChIP-quality p300 monoclonal antibodies. *Sci. Rep.* **4**, 5152 (2014).
22. Zhang, T. *et al.* Covalent targeting of remote cysteine residues to develop CDK12 and CDK13 inhibitors. *Nat. Chem. Biol.* **12**, 876–884 (2016).
23. Hollenhorst, P. C. *et al.* DNA specificity determinants associate with distinct transcription factor functions. *PLoS Genet.* **5**, e1000778 (2009).
24. Hnisz, D. *et al.* Super-enhancers in the control of cell identity and disease. *Cell* **155**, 934–947 (2013).
25. Oler, A. J. *et al.* Human RNA polymerase III transcriptomes and relationships to Pol II promoter chromatin and enhancer-binding factors. *Nat. Struct. Mol. Biol.* **17**, 620–628 (2010).
26. Navarro, J.-M. *et al.* Site- and allele-specific polycomb dysregulation in T-cell leukaemia. *Nat. Commun.* **6**, 6094 (2015).
27. Andricovich, J., Kai, Y., Peng, W., Foudi, A. & Tzatsos, A. Histone demethylase KDM2B regulates lineage commitment in normal and malignant hematopoiesis. *J. Clin. Invest.* **126**, 905–920 (2016).
28. Sanda, T. *et al.* Core transcriptional regulatory circuit controlled by the TAL1 complex in human T cell acute lymphoblastic leukemia. *Cancer Cell* **22**, 209–221 (2012).
29. Mansour, M. R. *et al.* Oncogene regulation. An oncogenic super-enhancer formed through somatic mutation of a noncoding intergenic element. *Science* **346**, 1373–1377 (2014).
30. Thurman, R. E. *et al.* The accessible chromatin landscape of the human genome. *Nature* **489**, 75–82 (2012).
31. Robson, M. I. *et al.* Constrained release of lamina-associated enhancers and genes from the nuclear envelope during T-cell activation facilitates their association in chromosome compartments. *Genome Res.* **27**, 1126–1138 (2017).
32. Kim, D. Y. *et al.* CBFβ stabilizes HIV Vif to counteract APOBEC3 at the expense of RUNX1 target gene expression. *Mol. Cell* **49**, 632–644 (2013).
33. Benyoucef, A. *et al.* UTX inhibition as selective epigenetic therapy against TAL1-driven T-cell acute lymphoblastic leukemia. *Genes Dev.* **30**, 508–521 (2016).
34. Orlando, D. A. *et al.* Quantitative ChIP-Seq normalization reveals global modulation of the epigenome. *Cell Rep.* **9**, 1163–1170 (2014).
35. Ip, J. Y. *et al.* Global impact of RNA polymerase II elongation inhibition on alternative splicing regulation. *Genome Res.* **21**, 390–401 (2011).
36. Hnisz, D. *et al.* Activation of proto-oncogenes by disruption of chromosome neighborhoods. *Science* **351**, 1454–1458 (2016).
37. Saint-André, V. *et al.* Models of human core transcriptional regulatory circuitries. *Genome Res.* **26**, 385–396 (2016).
38. Cuddapah, S. *et al.* Global analysis of the insulator binding protein CTCF in chromatin

barrier regions reveals demarcation of active and repressive domains. *Genome Res.* **19**, 24–32 (2009).

39. Seo, J. *et al.* Genome-wide profiles of H2AX and γ-H2AX differentiate endogenous and exogenous DNA damage hotspots in human cells. *Nucleic Acids Res.* **40**, 5965–5974 (2012).
40. Weintraub, A. S. *et al.* YY1 Is a Structural Regulator of Enhancer-Promoter Loops. *Cell* **171**, 1573–1588.e28 (2017).
41. Khan, A. & Zhang, X. dbSUPER: a database of super-enhancers in mouse and human genome. *Nucleic Acids Res.* **44**, D164–71 (2016).
42. Heinz, S. *et al.* Simple combinations of lineage-determining transcription factors prime cis-regulatory elements required for macrophage and B cell identities. *Mol. Cell* **38**, 576–589 (2010).
43. Lieberman-Aiden, E. *et al.* Comprehensive mapping of long-range interactions reveals folding principles of the human genome. *Science* **326**, 289–293 (2009).
44. Imakaev, M. *et al.* Iterative correction of Hi-C data reveals hallmarks of chromosome organization. *Nat. Methods* **9**, 999–1003 (2012).
45. Rao, S. S. P. *et al.* A 3D Map of the Human Genome at Kilobase Resolution Reveals Principles of Chromatin Looping. *Cell* **159**, 1665–1680 (2014).
46. Brady, T. *et al.* Integration target site selection by a resurrected human endogenous retrovirus. *Genes Dev.* **23**, 633–642 (2009).
47. Berry, C., Hannenhalli, S., Leipzig, J. & Bushman, F. D. Selection of target sites for mobile DNA integration in the human genome. *PLoS Comput. Biol.* **2**, e157 (2006).
48. ENCODE Project Consortium. An integrated encyclopedia of DNA elements in the human genome. *Nature* **489**, 57–74 (2012).
49. Love, M. I., Huber, W. & Anders, S. Moderated estimation of fold change and dispersion for RNA-seq data with DESeq2. *Genome Biol.* **15**, 550 (2014).
50. Love, M. I., Anders, S., Kim, V. & Huber, W. RNA-Seq workflow: gene-level exploratory analysis and differential expression. *F1000Res.* **4**, 1070 (2015).
51. Bray, N. L., Pimentel, H., Melsted, P. & Pachter, L. Near-optimal probabilistic RNA-seq quantification. *Nat. Biotechnol.* **34**, 525–527 (2016).
