# Supplementary material for: Spatially clustered loci with multiple enhancers are frequent targets of HIV-1 integration
Source: Nat Commun. 2019 Sep 6;10:4059. doi: 10.1038/s41467-019-12046-3 (PMC6731298; doi:10.1038/s41467-019-12046-3)
Supplement: Supplementary file 3 — Description of Additional Supplementary Files [file 41467_2019_12046_MOESM3_ESM.pdf]

Description of Additional Supplementary Files

File Name: Supplementary Data 1

Description: List of genes targeted by HIV-1
