# Supplementary material for: Spatially clustered loci with multiple enhancers are frequent targets of HIV-1 integration
Source: Nat Commun. 2019 Sep 6;10:4059. doi: 10.1038/s41467-019-12046-3 (PMC6731298; doi:10.1038/s41467-019-12046-3)
Supplement: Supplementary file 5 — Reporting Summary [file 41467_2019_12046_MOESM5_ESM.pdf]

nature research

Corresponding author(s): Marina Lusic

Last updated by author(s): Jul 18, 2019

# Reporting Summary

Nature Research wishes to improve the reproducibility of the work that we publish. This form provides structure for consistency and transparency in reporting. For further information on Nature Research policies, see Authors & Referees and the Editorial Policy Checklist.

## Statistics

For all statistical analyses, confirm that the following items are present in the figure legend, table legend, main text, or Methods section.

| n/a | Confirmed | |
|---|---|---|
| ☐ | ☒ | The exact sample size (*n*) for each experimental group/condition, given as a discrete number and unit of measurement |
| ☐ | ☒ | A statement on whether measurements were taken from distinct samples or whether the same sample was measured repeatedly |
| ☐ | ☒ | The statistical test(s) used AND whether they are one- or two-sided<br>*Only common tests should be described solely by name; describe more complex techniques in the Methods section.* |
| ☐ | ☒ | A description of all covariates tested |
| ☐ | ☒ | A description of any assumptions or corrections, such as tests of normality and adjustment for multiple comparisons |
| ☐ | ☒ | A full description of the statistical parameters including central tendency (e.g. means) or other basic estimates (e.g. regression coefficient) AND variation (e.g. standard deviation) or associated estimates of uncertainty (e.g. confidence intervals) |
| ☐ | ☒ | For null hypothesis testing, the test statistic (e.g. *F*, *t*, *r*) with confidence intervals, effect sizes, degrees of freedom and *P* value noted<br>*Give P values as exact values whenever suitable.* |
| ☒ | ☐ | For Bayesian analysis, information on the choice of priors and Markov chain Monte Carlo settings |
| ☒ | ☐ | For hierarchical and complex designs, identification of the appropriate level for tests and full reporting of outcomes |
| ☐ | ☒ | Estimates of effect sizes (e.g. Cohen's *d*, Pearson's *r*), indicating how they were calculated |

*Our web collection on statistics for biologists contains articles on many of the points above.*

## Software and code

Policy information about availability of computer code

Data collection: GSE122735; GSE122826; GSE122958; GSE134382

Data analysis:
https://github.com/gui11aume/genome_structure_and_HIV_integration/blob/master/maja/Brady_Integration_Sites.md
https://github.com/gui11aume/genome_structure_and_HIV_integration/blob/master/maja/is.Robj
https://github.com/gui11aume/genome_structure_and_HIV_integration/raw/master/maja/Replicates.RDS
https://github.com/ezorita/hi.c
https://github.com/mirnylab/cooler
https://github.com/G100DKFZ/gene-is
graphpad prism 6
volocity 6.3
FlowJo

For manuscripts utilizing custom algorithms or software that are central to the research but not yet described in published literature, software must be made available to editors/reviewers. We strongly encourage code deposition in a community repository (e.g. GitHub). See the Nature Research guidelines for submitting code & software for further information.

## Data

Policy information about availability of data

All manuscripts must include a data availability statement. This statement should provide the following information, where applicable:

- Accession codes, unique identifiers, or web links for publicly available datasets
- A list of figures that have associated raw data
- A description of any restrictions on data availability

All relevant data supporting the key findings of this study are available within the article and its Supplementary Information files or from the corresponding author on reasonable request. The RNA- Seq of resting and activated CD4+ T cells is available from Gene Expression Omnibus(GEO; Lucic et al GSE122735), ChIP-Seq on

Primary CD4+ T cells (Lucic et al. GSE GSE122826), in situ Hi-C data (Chen et al GSE122958). Integration site raw data on in vitro infected CD4+ T cells will be available from GSE134382.

# Field-specific reporting

Please select the one below that is the best fit for your research. If you are not sure, read the appropriate sections before making your selection.

☒ Life sciences ☐ Behavioural & social sciences ☐ Ecological, evolutionary & environmental sciences

For a reference copy of the document with all sections, see nature.com/documents/nr-reporting-summary-flat.pdf

# Life sciences study design

All studies must disclose on these points even when the disclosure is negative.

| | |
|---|---|
| Sample size | For HIV-1 integration sites in primary CD4+ T cells infected in vitro, we obtained 1475 uniquely mappable integration sites in activated cells |
| Data exclusions | We used only unique integrations from each study. |
| Replication | ChIP Seq data were performed in duplicates or triplicates, RNA Seq were performed in Triplicates. |
| Randomization | In order to assess if there is an enrichment of various chromatin features on sites of HIV-1 integration, we adapted the ROC curve areas method. The strategy was to use “nested case controls” - a collection of integration sites sampled from the genome which would act as control sites and can be compared to true integration sites. |
| Blinding | Allele counting and distribution in Volocity was performed by 2 different persons |

# Reporting for specific materials, systems and methods

We require information from authors about some types of materials, experimental systems and methods used in many studies. Here, indicate whether each material, system or method listed is relevant to your study. If you are not sure if a list item applies to your research, read the appropriate section before selecting a response.

## Materials & experimental systems

| n/a | Involved in the study | |
|---|---|---|
| ☐ | ☒ | Antibodies |
| ☐ | ☒ | Eukaryotic cell lines |
| ☒ | ☐ | Palaeontology |
| ☒ | ☐ | Animals and other organisms |
| ☒ | ☐ | Human research participants |
| ☒ | ☐ | Clinical data |

## Methods

| n/a | Involved in the study | |
|---|---|---|
| ☐ | ☒ | ChIP-seq |
| ☐ | ☒ | Flow cytometry |
| ☐ | ☐ | MRI-based neuroimaging |

## Antibodies

| | |
|---|---|
| Antibodies used | Abcam: H3K27ac (ab4729), H3K4me3 (ab8580) H3K36me3 (ab9050), H4K20me1 (ab9051), H3K9me2 (ab1220) IgG Rabbit (ab46540) , lamin B1(ab16048) , From Santa Cruz myc (9E10-sc-40) from Sigma Beta-Actin (AC-74)<br>Secondaray antibodies from Thermo Fisher, anti rabbit Alexa 488, 568, 647 |
| Validation | H3K27acetyl K27 (ab4729 ) ChIP grade (59 reviews) and 865 specific references, available on Abcam website<br>H3K4me3 (ab8580) ChIP grade, 1306 references available on Abcam website<br>H3K36me3 (ab9050) ChIP grade, tested in immunoflourescence . ChIP and ChIP seq 600 references, data sheet available on Abcam website<br>H4K20me1 (ab9051) ChIP grade, 90 references data sheet available on Abcam website<br>H3K9me2 (ab1220) ChIP grade, 580 references data sheet available on Abcam website |

## Eukaryotic cell lines

Policy information about cell lines

| | |
|---|---|
| Cell line source(s) | Jurkat, obtained from the cell collection of the Center for Genomic Regulation, Barcelona |

| | |
|---|---|
| Authentication | *Describe the authentication procedures for each cell line used OR declare that none of the cell lines used were authenticated.* |
| Mycoplasma contamination | none |
| Commonly misidentified lines (See ICLAC register) | *Name any commonly misidentified cell lines used in the study and provide a rationale for their use.* |

# ChIP-seq

## Data deposition

☒ Confirm that both raw and final processed data have been deposited in a public database such as GEO.

☒ Confirm that you have deposited or provided access to graph files (e.g. BED files) for the called peaks.

| | |
|---|---|
| Data access links *May remain private before publication.* | GSE122826 |
| Files in database submission | GSM3486205 Input 1<br>GSM3486206 Input 2<br>GSM3486207 Input 3<br>GSM3486208 Input 4<br>GSM3486209 Input 5<br>GSM3486210 H3K27ac_bio1_1<br>GSM3486211 H3K27ac_bio2_1<br>GSM3486212 H3K27ac_bio1_2<br>GSM3486213 H3K27ac_bio2_2<br>GSM3486214 H3K36me3_bio1<br>GSM3486215 H3K36me3_bio2<br>GSM3486216 H3K4me3_bio1<br>GSM3486217 H3K4me3_bio2<br>GSM3486218 H3K4me3_bio3<br>GSM3486219 H3K9me2_bio1<br>GSM3486220 H3K9me2_bio2<br>GSM3486221 H3K9me2_bio3<br>GSM3486222 H4K20me1_bio1<br>GSM3486223 H4K20me1_bio2 |
| Genome browser session (e.g. UCSC) | *Provide a link to an anonymized genome browser session for "Initial submission" and "Revised version" documents only, to enable peer review. Write "no longer applicable" for "Final submission" documents.* |

## Methodology

| | |
|---|---|
| Replicates | 2 or 3 |
| Sequencing depth | Illumina Hiseq 2000, 50 SE, with de-Multiplexing |
| Antibodies | H3K27acetyl K27 (ab4729 ); H3K4me3 (ab8580); H3K36me3 (ab9050); H4K20me1 (ab9051); H3K9me2 (ab1220) |
| Peak calling parameters | ChIP-Seq reads were mapped to human genome (GRCh37) using Bbmap with parameters minid=0.98, qtrim=lr, minavgquality=20. Resulting bam files belonging to same experiments were merged and sorted using bamtools. Average binding profiles in reads per million across sets of genes were made using ngsplot. Peaks were called using MACS2 , for every data set versus its matching input, with parameters --broad --broad-cutoff 0.1 -p 1e-9 -g 2.7e9 -B. All results were transformed to RPKM for downstream analysis and visualization<br><br>For Jurkat cells, ChIP-Seq reads were mapped to hg19 using BWA-mem. BWA options were as follows: '-k17 -r1.3 -B2 -O4 -T22' for read lengths less or equal to 30 nt, '-k18 -B3 -O5 -T28' for read lengths less or equal to 40 nt and default options for longer reads |
| Data quality | ChIP-Seq enriched regions were discretized using Zerone 17 with mapping quality cutoff 20 and enrichment confidence 0.99. |
| Software | Bpmap MACS2 |

## Flow Cytometry

### Plots

Confirm that:

☒ The axis labels state the marker and fluorochrome used (e.g. CD4-FITC).

☒ The axis scales are clearly visible. Include numbers along axes only for bottom left plot of group (a 'group' is an analysis of identical markers).

☒ All plots are contour plots with outliers or pseudocolor plots.

☐ A numerical value for number of cells or percentage (with statistics) is provided.

### Methodology

| | |
|---|---|
| Sample preparation | 150 000 were fixed in 3% paraformaldehyde for 10 min at room temperature. Cells were washed in 1% FBS/PBS and stained with corresponding antibody for 45 minutes on ice |
| Instrument | BD FACSVerse Instrument |
| Software | FlowJo software |
| Cell population abundance | CD69 marker detected 63.3% of positive cells 20hrs post stimulation with CD3/CD28 activating beads, whereas CD25 antibody detected 49% of cells as positive. Longer activation times (48hrs) resulted in more than 90% of cellular population positive for CD69 or CD25 markers. |
| Gating strategy | Gating strategy used to determine non activated (upper right panels) and activated cells (lower panels) with two markers, CD 69 (A) and CD25(B). Population of live unstained activated cells was used to set the gates: side scatter plus specific marker was used for activated T cell population identification. |

☒ Tick this box to confirm that a figure exemplifying the gating strategy is provided in the Supplementary Information.

## Magnetic resonance imaging

### Experimental design

| | |
|---|---|
| Design type | *Indicate task or resting state; event-related or block design.* |
| Design specifications | *Specify the number of blocks, trials or experimental units per session and/or subject, and specify the length of each trial or block (if trials are blocked) and interval between trials.* |
| Behavioral performance measures | *State number and/or type of variables recorded (e.g. correct button press, response time) and what statistics were used to establish that the subjects were performing the task as expected (e.g. mean, range, and/or standard deviation across subjects).* |

### Acquisition

| | |
|---|---|
| Imaging type(s) | *Specify: functional, structural, diffusion, perfusion.* |
| Field strength | *Specify in Tesla* |
| Sequence & imaging parameters | *Specify the pulse sequence type (gradient echo, spin echo, etc.), imaging type (EPI, spiral, etc.), field of view, matrix size, slice thickness, orientation and TE/TR/flip angle.* |
| Area of acquisition | *State whether a whole brain scan was used OR define the area of acquisition, describing how the region was determined.* |

Diffusion MRI ☐ Used ☐ Not used

### Preprocessing

| | |
|---|---|
| Preprocessing software | *Provide detail on software version and revision number and on specific parameters (model/functions, brain extraction, segmentation, smoothing kernel size, etc.).* |
| Normalization | *If data were normalized/standardized, describe the approach(es): specify linear or non-linear and define image types used for transformation OR indicate that data were not normalized and explain rationale for lack of normalization.* |
| Normalization template | *Describe the template used for normalization/transformation, specifying subject space or group standardized space (e.g. original Talairach, MNI305, ICBM152) OR indicate that the data were not normalized.* |
| Noise and artifact removal | *Describe your procedure(s) for artifact and structured noise removal, specifying motion parameters, tissue signals and physiological signals (heart rate, respiration).* |

Volume censoring | *Define your software and/or method and criteria for volume censoring, and state the extent of such censoring.*

## Statistical modeling & inference

Model type and settings | *Specify type (mass univariate, multivariate, RSA, predictive, etc.) and describe essential details of the model at the first and second levels (e.g. fixed, random or mixed effects; drift or auto-correlation).*

Effect(s) tested | *Define precise effect in terms of the task or stimulus conditions instead of psychological concepts and indicate whether ANOVA or factorial designs were used.*

Specify type of analysis: ☐ Whole brain ☐ ROI-based ☐ Both

Statistic type for inference
(See Eklund et al. 2016) | *Specify voxel-wise or cluster-wise and report all relevant parameters for cluster-wise methods.*

Correction | *Describe the type of correction and how it is obtained for multiple comparisons (e.g. FWE, FDR, permutation or Monte Carlo).*

## Models & analysis

| n/a | Involved in the study |
|---|---|
| ☐ | ☒ Functional and/or effective connectivity |
| ☒ | ☐ Graph analysis |
| ☐ | ☒ Multivariate modeling or predictive analysis |

Functional and/or effective connectivity

We used logistic regression to model the HIV-1 insertion landscape in Jurkat cells, based on the following four predictors: gene expression, distance of the gene to the closest super enhancer, sub-compartment of the gene and gene size.
The models were trained with the standard parameters of the glm function in R.

Multivariate modeling and predictive analysis

The models were trained with the standard parameters of the glm function in R. We used 5-fold cross validation to test combinations of transforms (hyperbolic arcsine and logarithmic functions) and / or discretization in quantiles. The best cross-validation scores were obtained after discretizing the predictors in combinations of tertiles and quartiles, so models I and II were trained on discretized variables (the same discretization was used for both models). We then removed one of the four variables, retrained the model in the same conditions and measured the probability that a gene is classified as HIV-1 target (typical targets in model I, hotspots in model II) given that it is indeed an HIV-1 target.
