# Supplementary material for: Spatially clustered loci with multiple enhancers are frequent targets of HIV-1 integration
Source: Nat Commun. 2019 Sep 6;10:4059. doi: 10.1038/s41467-019-12046-3 (PMC6731298; doi:10.1038/s41467-019-12046-3)
Supplement: Supplementary file 6 — Source Data [file 41467_2019_12046_MOESM6_ESM.zip › Source Data/Source data Supplementary figure 2.zip!/Source data Supplementary figure 2/Supplementary 2A/MYCandACTIN WB_39_40_Lucic et al.pdf]

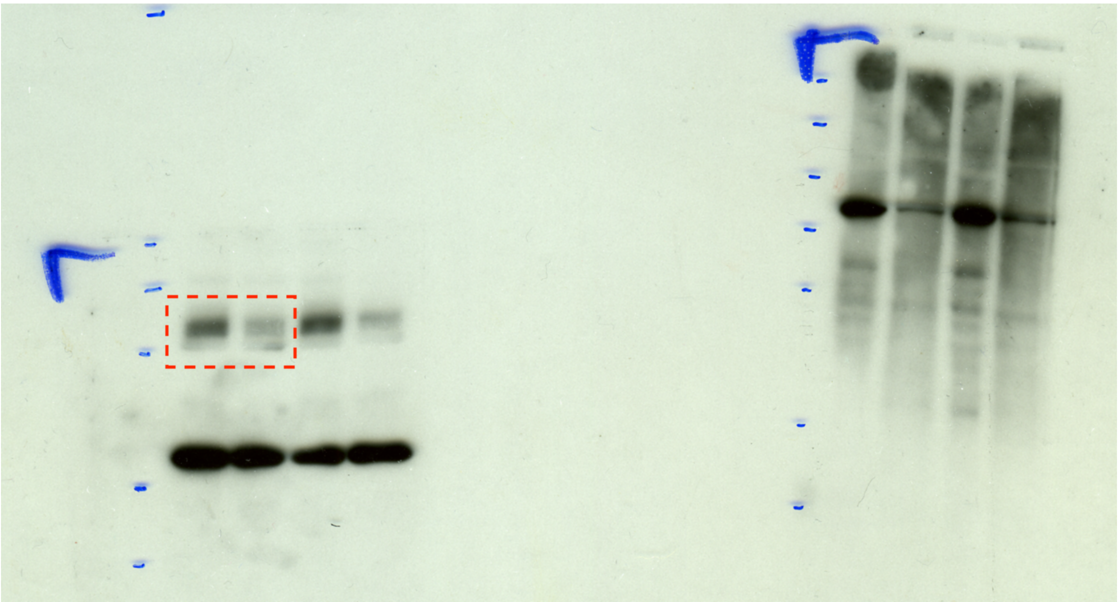

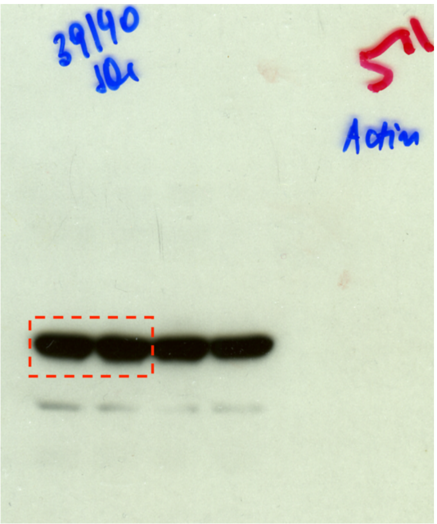
39/40
Actin
